# *What would you like to print*? Students' opinions on the use of 3D printing technology in medicine

**DOI:** 10.1371/journal.pone.0230851

**Published:** 2020-04-02

**Authors:** Renata Wilk, Wirginia Likus, Andrzej Hudecki, Marita Syguła, Aleksandra Różycka-Nechoritis, Konstantinos Nechoritis

**Affiliations:** 1 Department of Anatomy, School of Health Sciences in Katowice, Medical University of Silesia, Katowice, Poland; 2 Łukasiewicz Research Network–Institute of Non-Ferrous Metals, Gliwice, Poland; East Carolina University Brody School of Medicine, UNITED STATES

## Abstract

**Background:**

Recent advances in 3D printing technology, and biomaterials are revolutionizing medicine. The beneficiaries of this technology are primarily patients, but also students of medical faculties. Taking into account that not all students have full, direct access to the latest advances in additive technologies, we surveyed their opinion on 3D printing and education in this area. The research aimed to determine what knowledge about the use of 3D printing technology in medicine, do students of medical faculties have.

**Methods:**

The research was carried out in the form of a questionnaire among 430 students of the Medical University of Silesia in Katowice (Poland) representing various fields of medicine and health sciences. The questions included in the survey analyzed the knowledge of the respondents for 3D printing technology and the opportunities it creates in medicine.

**Results:**

The results indicate that students do have knowledge about 3D printing obtained mainly from the internet. They would be happy to deepen their knowledge at specialized courses in this field. Students appreciated the value of 3D printing in order to obtain accurate anatomical models, helpful in learning. However, they do not consider the possibility of complete abandonment of human cadavers in the anatomy classes. Their knowledge includes basic information about current applications of 3D printing in medicine, but not in all areas. However, they have no ethical doubts regarding the use of 3D printing in any form. The vast majority of students deemed it necessary to incorporate information regarding 3D printing technology into the curriculum of different medical majors.

**Conclusion:**

This research is the first of its kind, which allows for probing students' knowledge about the additive technologies in medicine. Medical education should be extended to include issues related to the use of 3D printing for medical applications.

## Introduction

3D printing technology is one of the fastest growing techniques of producing three-dimensional objects [1–5]. It is uniquely suited for producing, individualized, low-copy-number products. The use of 3D printing in medicine was a natural consequence of its development [6–9]. In surgery, 3D printing allows for a better understanding of the operating field on which the surgeon will work during a complicated operation, as well as the creation of tools tailored individually to the patient's anatomical conditions and personalized implants [4, 10–20]. Sometimes the problem of the availability of equipment necessary to treat patients is related to its price. Comparison of the price of a brand stethoscope with the price of the printed stethoscope showed that the 3D printing could, in some instances, solve this problem. Especially in countries with low expenditure on health care. The latest trends in the use of 3D printing technology are based on the combination of printed material with (usually) autologous living cells creating artificial tissues and organs for transplantation [21, 22]. The materials used for 3D printing in medicine are metal powders (including titanium), biodegradable polymers, rubber, carbon fibers, ceramics or light-cured resin. Hydrophilic tissue-scaffold resembling materials are becoming available, which allow for printing of a tissue-resembling structures, or even parts of organs. Promising tests were carried out using liver cells and nerve cells. [5, 6, 21–24]. For the purpose of medical education, models printed in 3D technology facilitate easier learning about the structure of the human body, because they are modeled on real anatomical specimens, while allowing far superior durability. The 3D printing could in this case also eliminate some ethical concerns, especially religious ones regarding the use of the human body in anatomy classes. The examples of use of 3D printing in medicine are in use currently preoperative, personalized models that serve as an aid in the preparation for complicated surgeries, for example within the spine, as well as craniofacial or heart surgeries. This allows for a better preparation of the operator for possible difficulties that may occur during the procedure, which shortens its duration, reduces the risk of medical errors and accelerates patient’s convalescence [8, 19, 20, 25].

For the reasons mentioned above, people who will practice medicine in the future, should be aware of the possibilities of 3D printing technology. They should know what 3D printing technologies are available, and also for what we can use such printed objects. The question is, do they really know?

## Materials and methods

### Study group

The study group consisted of the students of Medical University of Silesia in Katowice (Poland). The questionnaire was anonymous and voluntary. Respondents were selected randomly. The respondents who participated in the survey were 430 students, including 342 (79.5%) women and 88 (20.5%) men (the average age 21,7 ± 2,4 year).

### Methodology of questionnaire surveys

The study was carried out once, using an anonymous authors’ questionnaire prepared in electronic form. The questionnaire was created in the form of the docs.google.com website using the Google form. An invitation to participate in the survey together with a link to the questionnaire was posted on the University's online forums and sent via e-mail to the active Student Science Clubs of the Medical University of Silesia in Katowice. The survey was conducted from December 2017 to May 2018.

When preparing the survey, the authors took into account recommendations published so far for conducting surveys [26], and in particular online surveys (CHERRIES) [27–29].

The questionnaire contained 17 original questions (the questionnaire is in S1 Appendix).

The first part of the survey concerned the characteristics of the respondents, and included questions about: gender, age, major and year of study.

In the second part of the survey, open and closed questions with the possibility of multiple choice were used. The questions concerned: general knowledge of 3D printing, knowledge about the materials used for 3D printing and its use for medical purposes. The respondents could give an example of what they would like to print and also suggest their opinion regarding the ethics of 3D printing in medicine and the safety of printed implants for the human body. Students were asked about their previous contact with models obtained through 3D printing technology, the willingness to participate in activities on such topics and the aspect of choosing a human preparation or 3D model for learning anatomy during classes.

The full questionnaire is included as appendix 1 (S1 Appendix). The questionnaire was voluntarily completed by students of the Medical University of Silesia in Katowice, which, beside the random selection, was the only criterion for the selection of participants. All fields of medicine-related studies were taken into account, also regardless if full-time or part-time.

Due to the questionnaire nature of the study, it has not required the consent of the Bioethics Committee of the Medical University of Silesia (decision No KNW/KB/155/19).

### Statistical analysis

The collected results were subjected to statistical analysis. For this purpose, descriptive statistics were calculated (means and percentages of the group). Analysis of questions from the first part of the survey covered the entire study group. If the answer for question "Did you hear about 3D printing technology in medicine?" was negative, the answers to the remaining questions from the second part of the survey were not included in the analysis.

The calculations were performed using the Statistica 12.0 StatSoft Inc. software.

## Results

### Characteristics of the study group

Among the students taking part in the survey, the most numerous group were people aged 19–24, mainly 1st year students. Women accounted for the majority of respondents, 79.5% of respondents, men 20.5%. Above gender proportions in the conducted survey stems largely from the fact that some fields of study, are dominated by women. For example: the percentage of women among students in 2017–2019 was as follows: midwifery 99–100%, nursing 94–97%, physiotherapy 71–79%, electroradiology 75–85%, and medical faculty 59–64%. Similar gender distribution could be found at other medical schools across Poland, and beyond. Students filled out the questionnaire voluntarily, we did not interfere within the study group during the survey. Therefore, our research can be considered a representative of a larger population.

**Fig 1:**
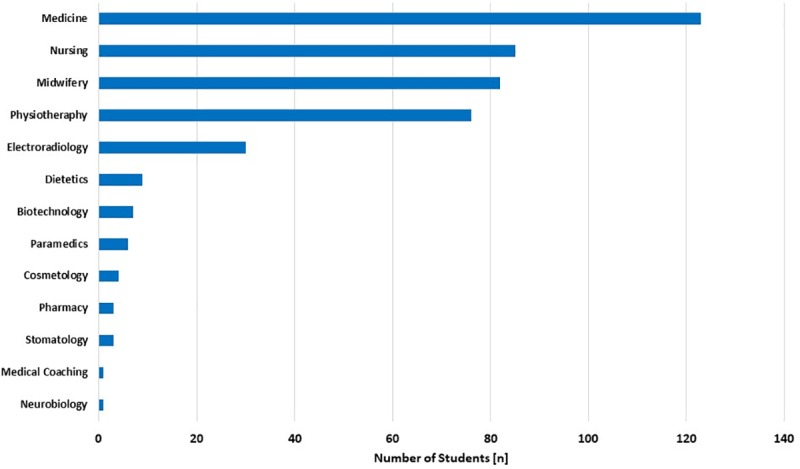
Distribution of students surveyed at the Medical University of Silesia according to the study field.

The largest share in the survey, according to the field of study, were students of the medical faculty (28.6%), subsequently: nursing, midwifery, physiotherapy and electroradiology (respectively: 19,8%; 19,1%; 16,67% and 7,0%). Directions: medical coaching and neurobiology were represented only by individuals (0.2% of respondents) (Fig 1).

**Fig 2:**
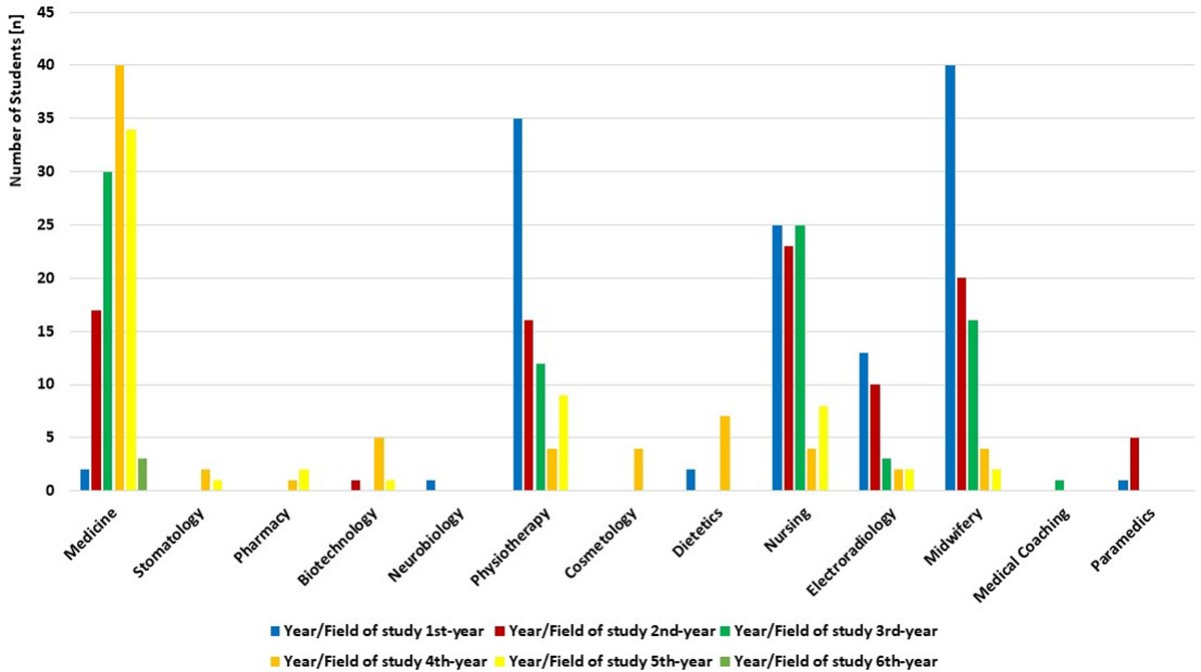
Characteristics of the study group according to the field and year of the study.

Analyzing the field and year of the study of the respondents, the largest group in the study were the students of the 1st year of midwifery and the 4th year of the medical faculty—40 persons each (9.3%). The smallest percentages of participants were recorded among students of medical coaching and neurobiology (one person from each study field) (Fig 2).

### Analysis of medical students' opinions on the use of 3D printing technology in medicine

The vast majority of respondents– 429 students heard about the use of 3D printing technology in the field of medicine. Only 0.2% of respondents, which means 1 person from the entire study group declared that he hadn't heard about the use of this technology in the medical field. This person was excluded from further analysis.

As the most frequent source of information about 3D printing, students pointed to the internet (67.6%). Among respondents, 18.4% declared that they obtained information about 3D printing from television. The lowest percentage of people gained knowledge about 3D printing from didactic classes and universities (respectively 0.7% and 0.5% of respondents) (Table 1).

**Table 1:** From what source do you most often gain the information about 3D printing technology? – students' opinions.

Source of information about 3D printing technology	Students–n (%)
**Internet**	290 (67,6%)
**Television**	79 (18,4%)
**Scientific periodicals**	25 (5,8%)
**Scientific Conferences**	16 (3,7%)
**Friends**	8 (1,9%)
**Didactic classes**	3 (0,7%)
**University**	2 (0,5%)
**Other**	6 (1,4%)

**Fig 3:**
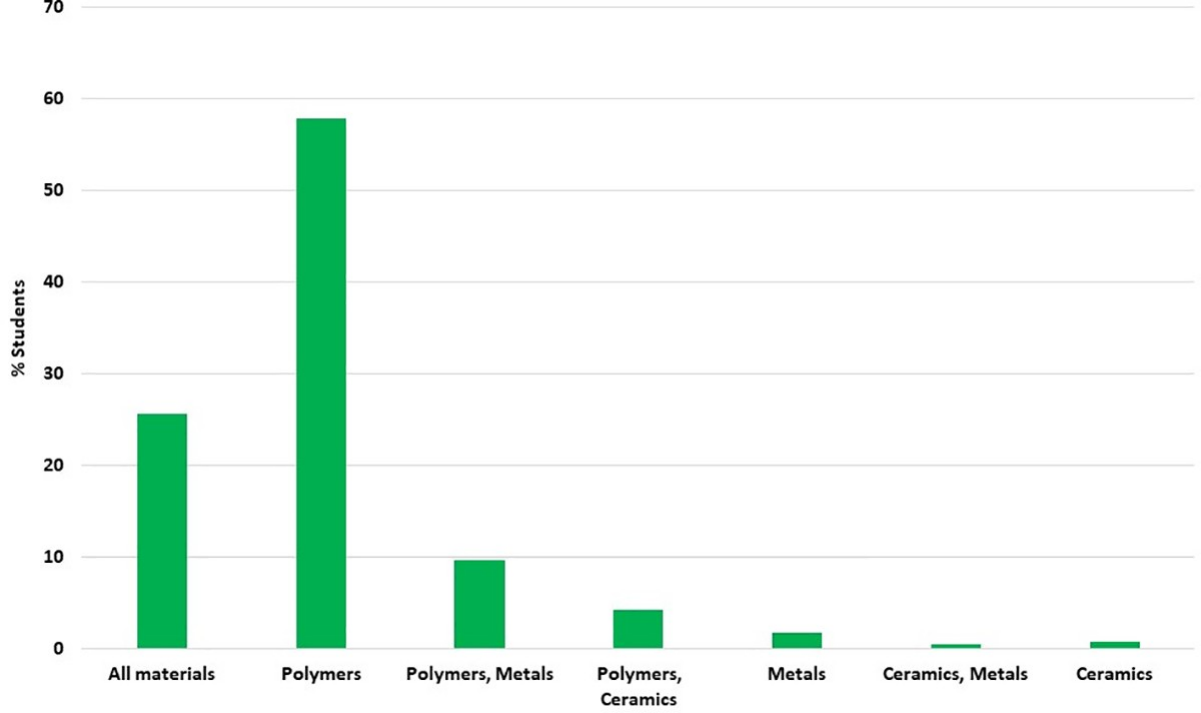
What materials are currently used in 3D printing technology? – students' opinions.

When asked about materials currently used in 3D printing technology, the respondents for the most part indicated polymer materials– 248 students. 110 students considered it possible to use all materials: polymeric, ceramic and metal materials listed in the survey. The least frequently mentioned material was ceramic (only 3 students indicated it as a single possible material, while in combination with metals 2 students, and with polymer materials 18 students). Polymers and metals as potential materials for 3D printing were indicated by 41 students (Fig 3).

**Fig 4:**
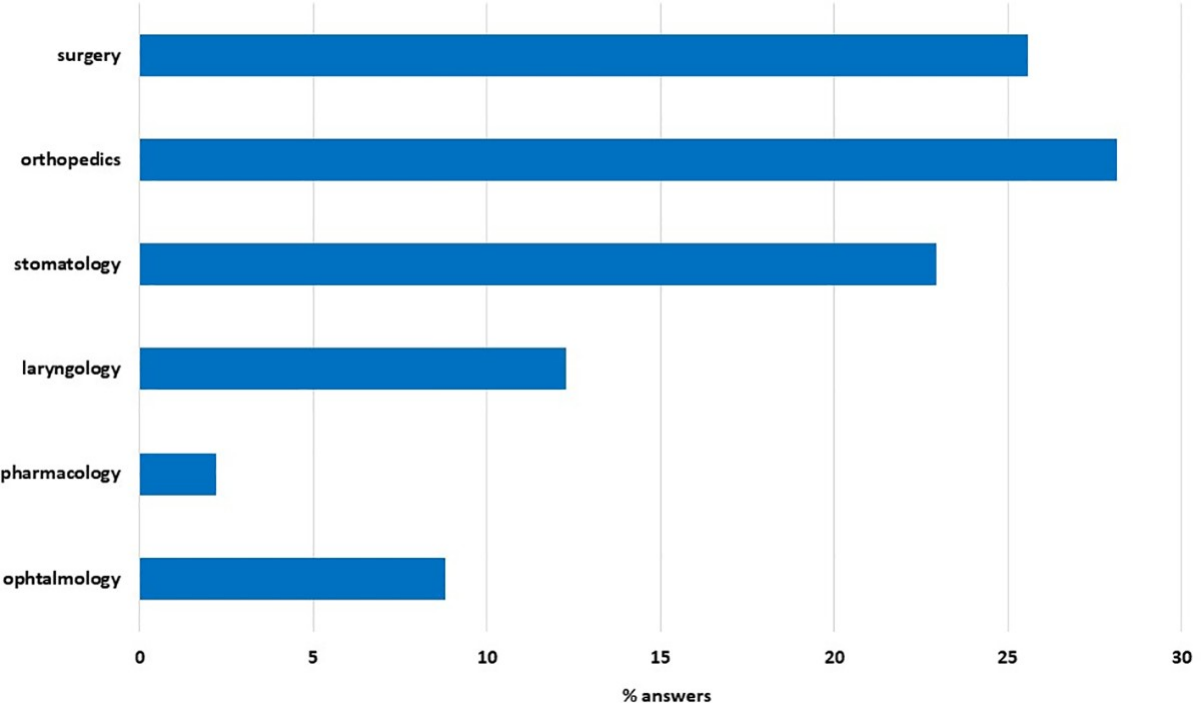
In what fields of medicine 3D printing is used most often? – students' opinions.

In the question about areas of medicine in which 3D printing is used most often, the respondents had the opportunity to choose from several fields. Summing up the individual answers, the most frequently marked area was orthopedics, which was indicated 395 times and accounted for 28.1% of all votes. Slightly less, 359 times (25.6%), general surgery was chosen as a field for the use of 3D printing technology. Among the responses, a significant number of votes—322 (22.9%) were also directed to dentistry. Subsequently, 173 times (12.3%) ENT and 124 times (8.8%) ophthalmology were indicated. The pharmacology was chosen least often among students, which accounted for 2.2% (31 votes) (Fig 4).

The most often chosen combination of the medical specialties used by the students were general surgery, orthopedics and dentistry– 103 students (23.9% of respondents) chose this combination. Other frequent connections were surgery and orthopedics (56 students, 13.0%); general surgery, orthopedics, dentistry, ophthalmology, ENT (this combination was chosen by 54 students, 12.8%) and surgery, orthopedics, dentistry, ENT (49 students, 11.4%).

Among the surveyed students, 268 (62.5%) were able to propose a practical application of 3D printing technology in medicine, under the condition that it would be possible. As an example, students listed mainly human organs such as: heart, liver, auricle, fibrous tissue in the form of ligaments, cartilage tissue and bone tissue as well as elements of the skull to supplement bone defects in case of its damage, upper and lower limbs prostheses. On the other hand, 37.5% (161) of the respondents were not able to indicate an example of the practical application of 3D printing technology in medicine.

Answering the question about the possibility of printing with the use of patient cells, 65.3% of the respondents (280 students) considered that there are such possibilities, while 34.7% (149 students) ruled out such a possibility.

Analysis of responses showed that only 142 participants of the survey (33.1%) had the opportunity to see a printed 3D model for medical purposes and a significant part, as much as 66.9% (287 students), admitted that they never saw objects printed with this technique that were intended for medical applications.

**Fig 5:**
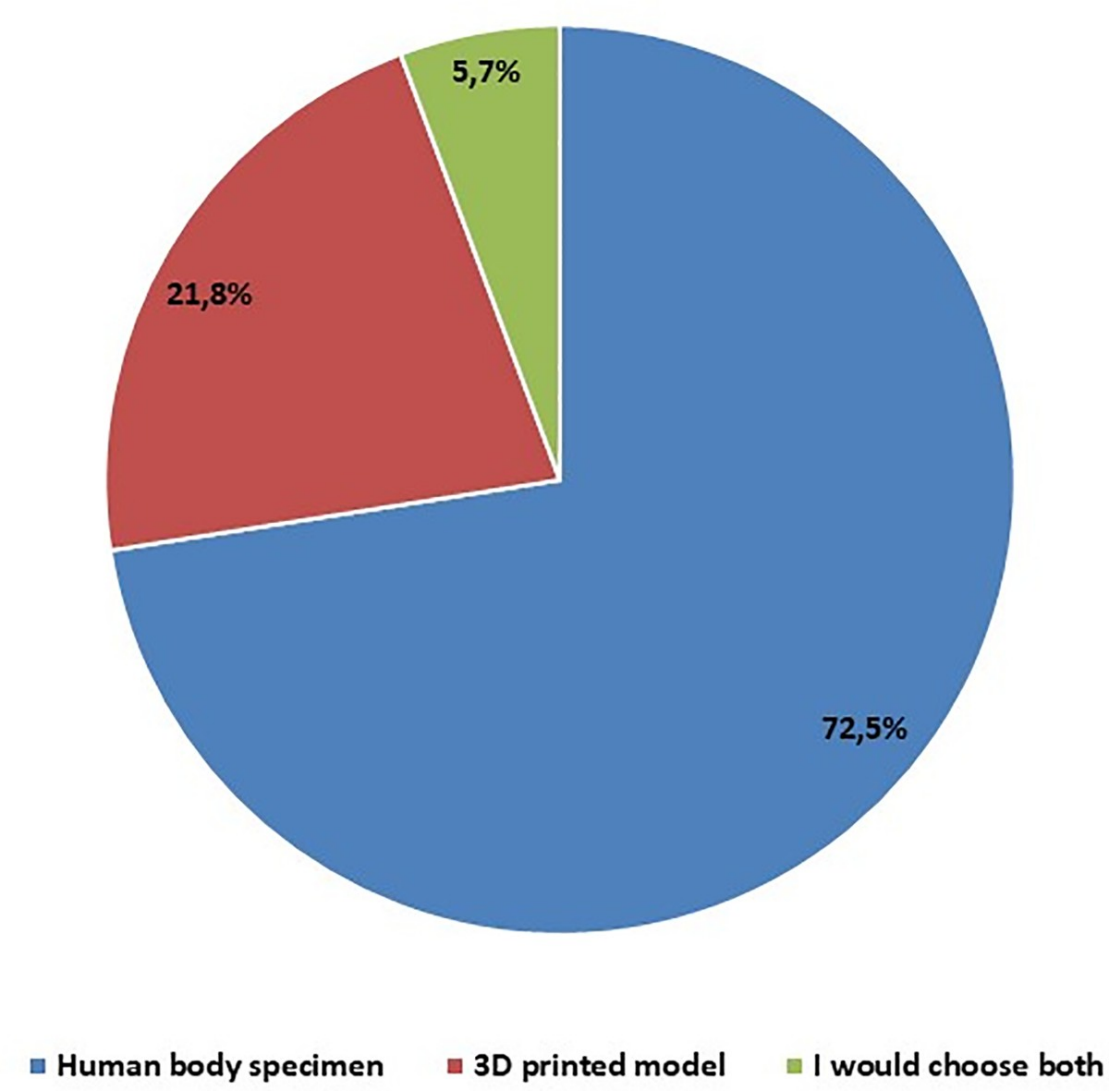
In the anatomy classes, with the choice of standard anatomical specimen (cadaver) and accurately mapped 3D model, what would you choose? – students' opinions.

Answers to the question about the choice of a "standard anatomical specimen—cadaver" or "accurately mapped 3D model" in the anatomy classes, were analyzed only from students who dealed with cadavers during their anatomy classes. The results of the analysis showed that the majority of students– 293 more willingly chose the commonly used option–cadavers. The group of 23 respondents did not rule out using the three-dimensional model simultaneously, considering both options to be beneficial for didactic purposes. "An accurately mapped 3D model" was indicated by 88 students (Fig 5). People who chose both options argued that they would like to compare the standard anatomical specimen with the model printed using the 3D printing method. One of the respondents, pointing to the advantage of the use of cadavers, justified that with such a specimen one is able to accurately feel the structure of tissues and observe the impact of the way of life on organs, e.g. lung damage for smokers and stomach damage by peptic ulcers. People who considered the 3D model as a better option to learn anatomy justified a better presentation of anatomical details than a standard anatomical specimen.

**Fig 6:**
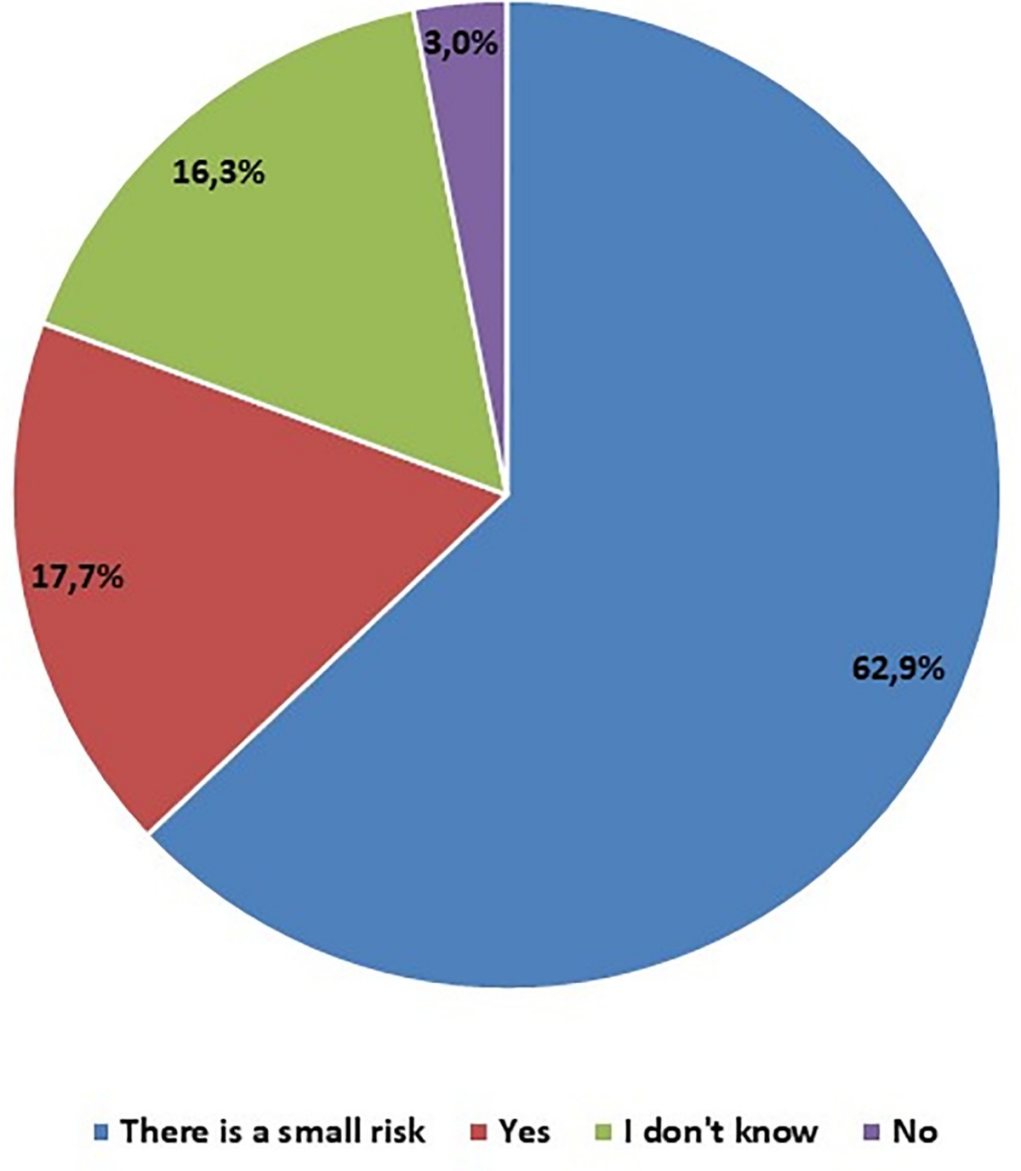
In your opinion, is printed in 3D object, implanted in the body, completely safe? – students' opinions.

Analyzing the answer to the question about the implant's safety for the human organism, 76 participants considered implanting the objects from 3D printer into the body as completely safe. Most, as many as 270 people, notice that there is some risk associated with the transplant, and only 13 students perceive the implant as dangerous to the body. 70 people did not give a definite answer–they chose the option "I don’t know" (Fig 6).

**Fig 7:**
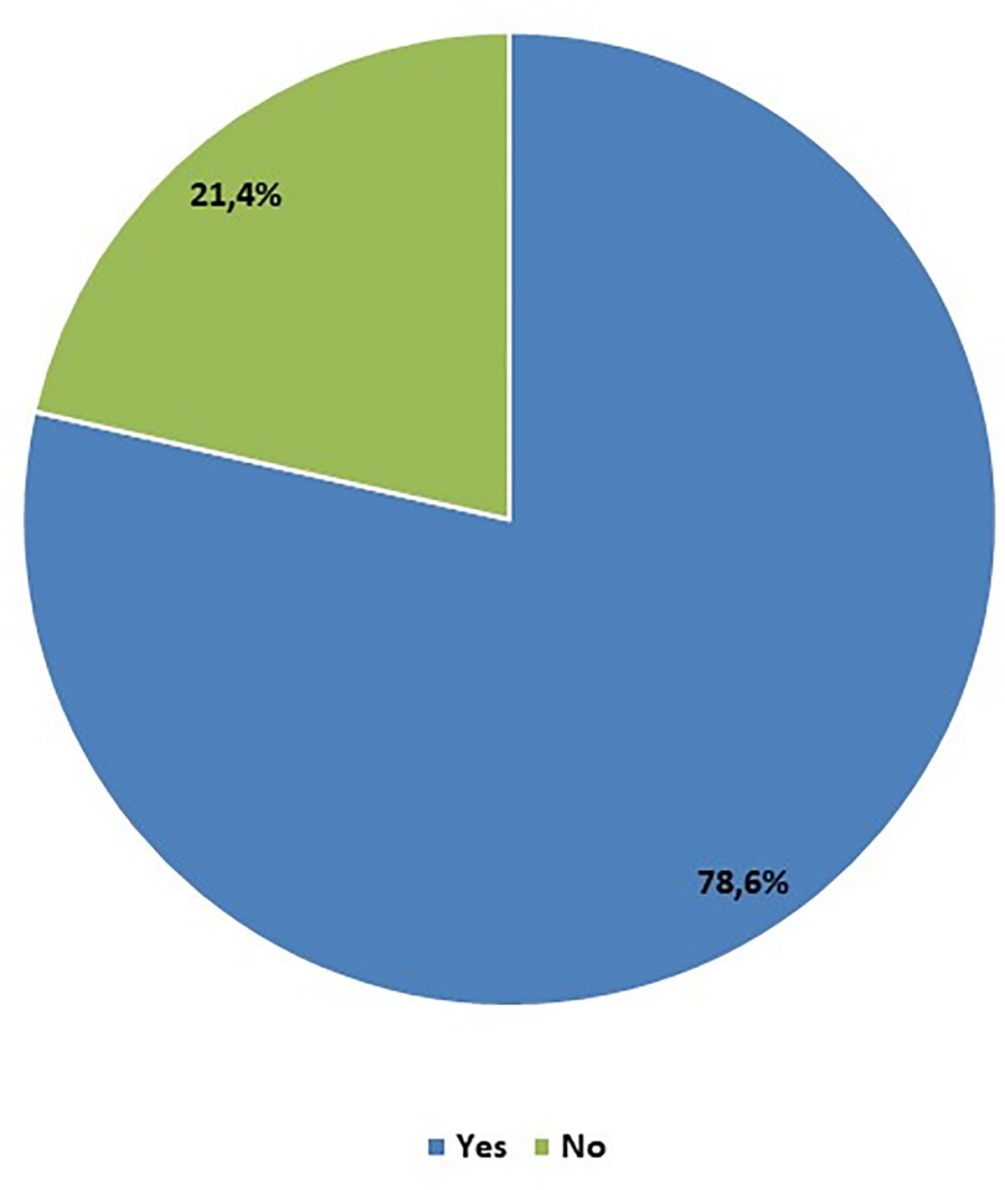
Do you think that printing organs is ethical? – students' opinions.

Students were also asked about the ethical issues of 3D printing. For most of them (349) printing organs is ethical, however 68 respondents said they do not have the opinion or knowledge on the subject. Only 12 respondents considered this behavior unethical (Fig 7).

**Fig 8:**
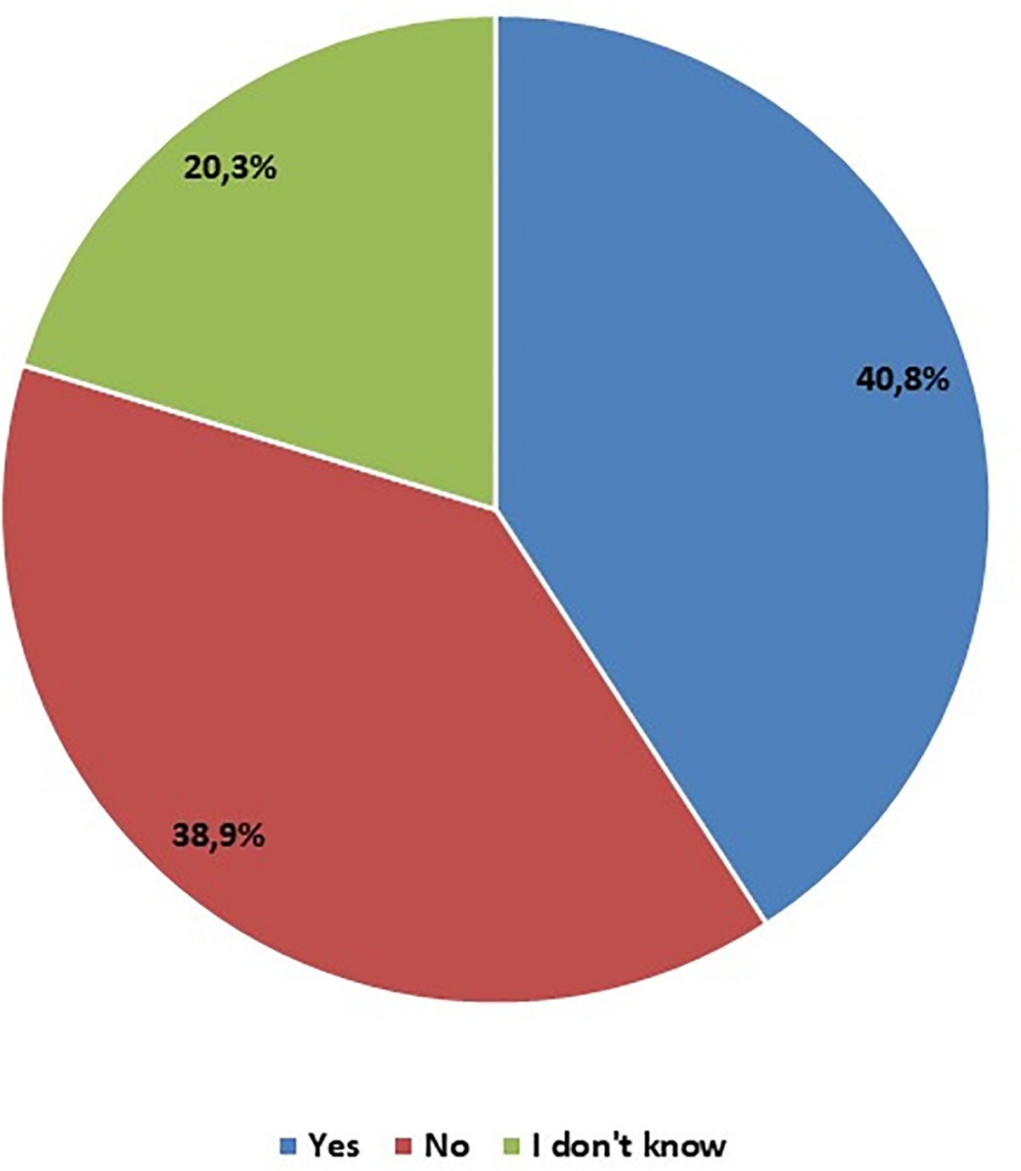
Regarding of the progress of biomedical engineering, do you think that any structure of the body can be printed in the future? – students' opinions.

Answering the question about possibility of printing every structure of the organism in future, taking into account the progress of biomedical engineering, 175 respondents believe that in the future it will be possible to print every structure of the organism. 87 stated that they have no knowledge on this subject. However, 167 students think that will not be possible to print all the structures of the body using 3D printing technologies (Fig 8).

The respondents, with a fully equipped 3D printing laboratory at their disposal, would most likely print the human heart. Most students also pointed to other internal organs of the human body, paying attention to the constant need of transplant organs. Among them there were specified: kidney, liver, pancreas and lungs. Students in their justifications were guided primarily by the needs of other people, patients or people from their family. As an example, many respondents gave bone, joint structures, and prostheses. For other applications, students would like to print anatomical models as educational help. Some respondents did not fully understand the current limitations of 3D printing technology. They pointed to the possibility of printing live organs or single cells such as T lymphocytes. This also shows that students see a great potential for 3D printing for the needs of medical science. A summary list of selected students’ proposals is presented in Table 2.

**Table 2:** Examples of 3D printouts indicated by Medical University of Silesia students.

Human internal organs	Bone and joint structures	Prostheses/ Implants	Other
**heart**	Bones	endoprostheses	anatomical model
**kidney**	bony elements	ear/nose prostheses	plaster dressing
**brain**	joint elements	bone implants	medical equipment
**liver**	Skull	parts of prostheses	heart valves
**pancreas**	Pelvis	orthodontic apparatus	tissue
**lungs**	Teeth	tooth implant	T lymphocytes
**eye**	knee joint		exoskeleton
**skin**	shoulder joint		optic nerve
**ear**	vertebral column		
**nose**	ligaments of the knee		
**stomach**			

Research has shown that students recognize the need to deepen their knowledge of 3D printing. Answering the question about the interest in deepening the knowledge about new technologies, 78.6% of students (337) expressed their willingness to participate in activities related to 3D printing, which is the answer to the question of the need to create seminars or lectures covering the above topic contained in the program of study of medical majors. 92 students (21.4%) do not feel the need to broaden their knowledge about modern technologies used in medicine during their studies.

## Discussion

The study on the knowledge and opinions regarding the use of 3D printing in medicine among students of medical university is the first study of this type carried out at medical universities in Poland. There are no similar surveys from other research centers in the world. In most cases, the respondents were women, because medical majors (especially such as midwifery or nursing) are overwhelmingly attended by females.

One of the areas of 3D printing applications is to use 3D printed models for teaching of anatomy. Studies on students`experiences related to the use of 3D printed models in the anatomy classes showed that multi-material and color 3D prints of upper limb models were considered by students to be sufficiently anatomically accurate for use in the classroom. Most students appreciated the availability of printed models compared to plastinates, and color prints also improved the efficiency of learning in their opinion [30]. However they pointed to some inaccuracies of the model especially to sense the differences between various types of tissues. In our research, students expressed interest in using models printed with 3D technology, they also did not rule out combining both human and three-dimensional models during classes but they would not be ready to completely abandon cadaveric specimens for 3D models. Similar conclusions were drawn by other authors dealing with these topics [31].

Acquiring bodies for scientific purposes is burdened with many ethical doubts, starting with the source of their acquisition and the use of formaldehyde to store them. The only ethical doubts regarding models from 3D printing that appeared in the opinions of concerned students, is the commercial use of preparations derived from voluntary donations through their copying by 3D printing [30, 31]. The study aimed at assessing the effectiveness of teaching anatomy using three-dimensional models was conducted among medical students using printed skull models. The study showed that, ultimately, better results were achieved by the group of students using 3D models for learning in comparison to those using atlases or even real skull [32]. In other studies, the effectiveness of external anatomy studies was analyzed. The research was carried out according to a similar pattern, included students using: 3D printed models, wet cadaveric specimens and combination of wet cadaveric specimens and 3D printed models. The results showed better achievements in the test obtained in the group working only with 3D models however it may be result from students' fears of getting in contact with human cadavers and consequently, inadequate use of the specimens. Another convenience for 3D preparations is the ability to prepare color prints, which, according to some authors [33], facilitates the absorption of material especially for new students. In heart anatomy learning analysis [20, 34] however, there was no significant advantage of models printed in 3D over standard models of the heart.

The effectiveness of 3D printing in learning anatomy has been demonstrated by the results of the research team from the University of Sussex. Based on the CT studies performed on bodies, 3D prints of anatomical structures of various parts of the body (mainly bones) were prepared in various colors and sizes. The models were available to students at the classes and also as take-home materials. Students using 3D printed models obtained higher results in tests checking their anatomical knowledge. Ethical doubts aroused with the option of sharing the files, used for individual printing of 3D models, through the internet in the form of free software. In students opinion this would not be consistent with the wish of donors [35]. These works indicate the need for further analyzes related to the use of models created in 3D printing technology in teaching anatomy in order to collect more data.

3D printing technology currently allows for combining different materials, e.g. rigid and hard as polylactic acid (PLA). To make the model more realistic bones should be printed using PLA, whereas muscles and mucous membranes with a flexible and soft material like FilaFlex3D [36]. In the Smith and Jones study [36], a model of larynx prepared in 3D printing technology, allowed for extension of muscles as well as to examine the action of internal muscles of the larynx on the vocal folds position change.

Currently, the biggest limitation for the widespread of use 3D printing technology is price, but innovations in this field should make it more accessible in the near future, e.g. for printing models on a printer These types of models would in fact be able to replace specimens derived from bodies of donors while eliminating problems with their availability as well as ethical or religious concerns related to their acquisition. According to the results of our research presented in the study, 3D printing did not raise ethical doubts among the surveyed students.

While teaching anatomy, and especially pathological anatomy, it is of great importance to seek anatomical variations in order to distinguish them from pathological changes. Thanks to 3D modeling, it is possible to preserve anatomical specimens of special educational values in the form of digital files and prevalence them among students. "Specimens" can also be created based on CT and MRI studies of living patients, which further extends the scope of their use as three-dimensional prints [37, 38].

Information about students' opinions on the use of 3D printing is valuable, because students and graduates of medical faculties can already meet 3D printing technology and its practical use in many places. For example, as part of professional training while advancing qualifications, 3D printing is proposed for use e.g. in the training of nurses [39]. In the research based on the recognition of heart defects, the obtained results indicated a better acquisition of knowledge concerning the pathology of this disease in a group of nurses having access to 3D printouts [40].

In surgical practice precisely printed surgical instruments [41] as well as preoperative models are already used e.g. in thoracic surgery or in interventional cardiology as a way to practice a course of a planned surgery [18, 42]. 3D printing technology can be used in preoperative planning and doctor training or i.e. for testing devices necessary in cardiac valves surgeries [43]. Similar in the case of complicated procedures such as TAVR (transcatheter aortic valve replacement) or LAA (left atrial appendage) occlusion, the possibility of carrying them out on a model with mapped anatomical conditions of the patient seems invaluable [44]. Difficult oncological surgeries, especially when the subsequent reconstructive surgery is required, are also exercised, in many cases, using the models of operational fields and adjacent structures printed in 3D technology [17].

Thanks to this technology we can also obtain accurate simulators faithfully reflecting the anatomical conditions used in the training of students and resident-doctors [45]. From patient`s point of view, the creation of individual 3D models also allows easier and more accurate understanding of his disease, which is important in the therapeutic process [46]. In addition to planning the procedure, 3D technology is already used to create individualized implants based on CT images, perfectly suited to the anatomical conditions of the patient. In the case of bone implants, such individual models allow to preserve all functions of the body part that they belong to and also by reducing pain, usually caused by pressure applied on nearby peripheral nerves, accelerate the patient's return to full efficiency [47].

Students' knowledge about the use of 3D printing especially in surgery, and in orthopedics, is quite high. These were the two highest-ranked medical specialties that students listed as proposals for possible use of 3D printing. Other two in the rank were laryngology and ophthalmology. In the case of physiotherapy, 3D printing is used to create individual orthosis recommended to patients especially during their rehabilitation or to restore their proper mechanics [10]. Based on surveys, it can be concluded that this aspect of 3D printing usage, as well as in dentistry, is well-known to the students. Especially in dental prosthetics in which 3D printing technology disseminated the creation of models necessary in prosthodontics or in maxillofacial surgery eliminating dental impressions or casts creation. Currently, printing of 3D models, individually matched to the patient, directly in a dentist's office is possible based on CT studies of individual patient [15].

There are other examples where 3D printing technology might be useful. Sometimes the problem of the availability of equipment necessary to examine and treat patients is associated with its price. Comparison of the price of a brand stethoscope with the price of a printed stethoscope showed that 3D printing could be a good solution. Such 3D printed stethoscope can be connected to a smartphone allowing a physician to consult patients at a distance. This aspect is especially important in countries with low expenditure on health care. [48–50].

Our results revealed that students often lack advanced knowledge about the bioprinting. Over half of the students surveyed are aware of possibility of 3D printing with the use of patient's cells, but they do not really know how such procedures are conducted from technical point of view. In proposals of elements that they proposed to print, appeared a single cell, a lymphocyte, which is currently not possible, and it is unlikely that it would become possible in a near future. Structures could be printed using whole cells, however single cell cannot be printed so far. 40.7% of respondents are convinced that in the future, thanks to the development of biomedical engineering, any structure of the organism, including entire organs, will be available through 3D printing technology. As organs best suited for 3D printing proposed by students is a heart, because as a single organ, without which it is impossible to live, it cannot be obtained otherwise than from the deceased donors. However, the mere printing of organs raises a question about the ethics and safety of using "printed" organs. Therefore, detailed regulations regarding 3D bioprints and implantation of "printed" implants or organs for people seem necessary.

Our study confirmed that students show interest in new technologies, especially those that expand the possibilities of personalized therapy and are innovative. Most of them know the advantages and limitations of 3D printing. Students see the need to use 3D printing in modern medicine in such areas as printing prostheses, organs, or creating models both as a teaching aid and pre-operational planning. The results of this work can be used by medical universities and other institutions responsible for medical education in order to determine the need for possible broadening the knowledge of students about possible use of this technology in their future profession. This knowledge may in the future result in gradual increase in the quality of medical care. Our research confirms that courses on 3D printing technology should be included in the education of people who decide to associate their future with one of medical professions, including future doctors, nurses and physiotherapists. Providing students with this knowledge will allow them in future to apply it independently in solving problems visible in patients in order to improve their comfort of life or accelerate the treatment process.

## Conclusions

1. Students have general knowledge about the use of 3D printing technology in medicine, which they obtained mostly from the internet. A small percentage of them obtained knowledge from scientific articles.
2. The results of the survey indicate that there is a great interest among students of medical majors on the latest achievements of 3D printing technology in medicine. Students are willing to continue to learn about biotechnological innovations.
3. Due to the dynamic development of technology, it seems important to consider the possibility of providing students with modern anatomical models printed in 3D printing technology during the anatomy classes to illustrate complex structures. It is optimal to combine a traditional form of classes with the use of cadavers and 3D models.
4. Most students can indicate an example of the possible use of a precise and realistic three-dimensional model in medical practice, taking into account the currently used materials.
5. The knowledge about the possibilities of using 3D printing in pharmacology, ophthalmology and otolaryngology (ENT) is currently not widely spread among medical students.
6. In the opinion of the respondents organ printing in 3D-technology meets ethical standards.

## Supporting information

S1 Appendix The questionnaire. (XLSX)
